# Impact of COVID-19 on essential healthcare services at the primary healthcare level in Armenia: a qualitative study

**DOI:** 10.1186/s12875-024-02377-6

**Published:** 2024-04-24

**Authors:** Varduhi Hayrumyan, Arpine Abrahamyan, Arusyak Harutyunyan, Lorky Libaridian, Serine Sahakyan

**Affiliations:** Turpanjian College of Health Sciences, American University of Armenia, 40 Baghramyan Ave, Yerevan, 0019 Armenia

**Keywords:** Essential health services, Primary healthcare, COVID-19, PHC service delivery, PHC service utilization, Disruptions, Telemedicine, Patient-provider communication, In-person visits, Vulnerable populations

## Abstract

**Background:**

The COVID-19 pandemic has presented significant global healthcare challenges, particularly impacting the continuity of essential health services in low- and middle-income countries. This study investigates the impact of the COVID-19 pandemic on the utilization and provision of essential health services in Armenia.

**Methods:**

We employed a conventional qualitative study design, conducting semi-structured in-depth interviews (*n* = 17) within public and private primary healthcare (PHC) facilities in Armenia in 2021. Our study participants encompassed physicians providing specialty services in PHC facilities (e.g. endocrinologists, gynecologists/obstetricians, and pediatricians), regular visitors to PHC facilities (e.g. adults with chronic diseases, parents of children), and policymakers. Thematic analysis was conducted, yielding five emergent categories: mobilization and organization of PHC services during COVID-19; PHC visits during COVID-19; worsening of chronic conditions due to the decline in PHC visits; problems with routine childhood vaccinations; and patient-provider communication challenges.

**Results:**

The number of in-person visits to PHC facilities declined due to adaptations in service delivery, imposed lockdown measures, and the public’s fear of visiting healthcare facilities. Maternal and child health services continued with no major disruptions. PHC providers deliberately limited the number of maternal and child visits to essential antenatal care, newborn screenings, and routine childhood immunizations. Still, children experienced some delays in vaccination administration. The pandemic resulted in a notable reduction in follow-up visits and monitoring of patients with chronic conditions, thereby exacerbating their chronic conditions. Phone calls were the primary method of patient-provider communication during the pandemic.

**Conclusions:**

The COVID-19 pandemic has had a profound impact on the delivery and utilization of essential healthcare services at PHC facilities, especially for those with chronic conditions who needed continuous care. Unified national-level guidance and technical capacity are needed to direct the provision of essential services at the PHC level, promote effective health communication, and implement digital platforms for the uninterrupted provision of essential care during public health emergencies.

**Supplementary Information:**

The online version contains supplementary material available at 10.1186/s12875-024-02377-6.

## Introduction

Essential health services were disrupted globally due to the Coronavirus Disease 2019 (COVID-19) pandemic. This disruption had the potential to result in adverse effects on individual and population health by negatively impacting preventable and treatable diseases [1–3]. During the pandemic, countries encountered various challenges and were obligated to make difficult decisions to maintain essential health services through strategic planning and coordinated actions [4]. 

Primary healthcare (PHC) is the cornerstone of healthcare systems, serving as the basis for universal health coverage; any disruption of services provided in this setting can be associated with a major impact on health outcomes and public health [5]. PHC plays a decisive role in the prevention and treatment of diseases, especially for vulnerable populations: children, older adults, and people living with chronic conditions and disabilities [6, 7]. Some PHC-oriented health systems displayed resilience, faster adaptation, and better maintenance of essential health services, while simultaneously taking on the responsibility of detecting and managing mild to moderate COVID-19 cases, and conducting triage to best manage hospital capacities [7]. Additionally, various successful reforms during the pandemic in the PHC sector guaranteed more successful COVID-19 responses in some countries, highlighting the importance of consistent and long-term investments in PHC for ensuring proper maintenance of essential health services in countries with comparable context [8]. To facilitate the continuity of PHC essential services, various adaptations and strategies were implemented: a shift to telehealth and community-based healthcare; limitation of patient-provider in-person encounters; adoption of public health measures; enhancement of human resource and surge capacities; improvement of the availability of and access to essential products and medications; and proactive targeting of vulnerable populations for providing necessary care [1, 9]. Despite its recognized critical role in addressing public health needs in emergency situations, PHC was often under-prioritized and underfunded by governments during the pandemic, especially in low- and middle-income countries (LMIC), resulting in disruptions in PHC services [8–11]. 

PHC essential services were one of the most affected by the pandemic: 53% of 80 countries participating in the World Health Organization (WHO) global pulse survey reported disruptions in PHC essential services [1]. A review involving 517 articles across 49 countries worldwide focusing on PHC service utilization reported that there was about a 56% decrease in in-person PHC services utilization due to the pandemic [12]. Thus, people continuously experienced reduced in-person care in PHC settings, which were only partially compensated for by remote consultations [1]. A combination of supply and demand factors are responsible for the disruption of services worldwide, including: lack of human resources and decreased availability of essential medications and other essential products; service delivery interruptions such as closures or rescheduling of services; and reduced care seeking by individuals due to fear of infection, lack of trust, financial difficulties and other challenges [1, 9]. 

Significant disruptions were observed in PHC essential services provided to patients living with chronic conditions such as cancer [13–16], cardiovascular diseases [16, 17], diabetes [16], chronic respiratory diseases [16] and other non-communicable diseases, leaving them at increased risk of severe illness from COVID-19, and worsened non-communicable diseases-related morbidity and mortality [1, 9–11]. Maternal and child healthcare services at PHC facilities were also adversely affected, leading to increased maternal and child mortality rates [18–20]. Routine immunizations were among the most effected PHC essential services due to the COVID-19 pandemic, raising concerns about the possibility of future outbreaks of vaccine-preventable diseases [1, 21]. According to the WHO global pulse survey (2021), which assessed 66 essential health services in more than 100 countries, disruptions were reported in about half of the services assessed (45%), with LMICs more affected than high-income countries [1]. 

Armenia is a middle-income country with 2.9 million population. The first case of COVID-19 in Armenia was detected on March 1, 2020. Since then, Armenia has experienced five COVID-19 waves [22]. As of July 27, 2023, there were 449 263 confirmed cases and 8 751 deaths [22]. In Armenia, the first COVID-19 vaccines became available for vulnerable and high-risk groups in April 2021, and soon thereafter became available to the general public [23, 24]. However, Armenia has been struggling to reach sufficient vaccine coverage against COVID-19. By the time of the study (mid-Summer 2021), only 4% of the population had received at least one dose of the vaccine [25, 26]. 

Since the beginning of the pandemic, COVID-19 testing was the responsibility of the National Center for Disease Control of Armenia and was conducted in designated testing and hospital facilities. In May 2020, the government of Armenia made the PHC sector responsible for the management and treatment of mild to moderate cases of COVID-19 [27]. The PHC sector’s responsibilities included contact tracing, sampling, sample transportation, treatment and follow-up of mild COVID-19 cases without complications, and coordination of hospitalization of patients with the triage center [27]. 

PHC services in Armenia are provided in approximately 340 facilities, including urban polyclinics, rural ambulatories and health centers across the country [28]. Armenia’s population is predominantly Armenian, accounting for 98.1%, with the remaining population consisting of minority groups including Yazidis, Russians, Kurds, Assyrians and others [29]. The entire population of Armenia, regardless of their nationality, is eligible to be served by the PHC sector; about 98% of the Armenian population is empaneled to PHC facilities and receives state-guaranteed health services [30]. According to a nationwide survey conducted in Armenia, females and those with higher monthly expenditures were more likely to avoid or delay routine medical care during the COVID-19 pandemic in PHC facilities. The same study showed, that other demographic characteristics such as nationality (Armenians vs. others), place of residence (rural vs. urban), education, and employment were not associated with avoidance or delay of routine, urgent, or any type of medical care [31]. PHC services in Armenia include disease prevention (including immunizations), specialist consultations, basic diagnostic examinations, chronic disease management (e.g. cardiovascular, endocrine diseases), and maternal and child health services [32]. PHC essential services are provided by subspecialists such as endocrinologists, cardiologists, gynecologists, and pediatricians, who often complement internists and family physicians in the PHC facilities in Armenia.

Throughout the country, PHC service utilization declined remarkably in 2020 (1 388 724 visits) compared to 2019 (2 055 102 visits) [28, 33]. According to experts, underutilization of PHC services, especially for chronic conditions such as cardiovascular and respiratory diseases, could lead to excess deaths in Armenia [28, 33]. One of the reasons for the underutilization of PHC services were the COVID-19 restrictions [28, 33]. Another reason for underutilization of services was the 44-day war with Azerbaijan, which caused displacement, injury and death of thousands of people in Armenia and diverted attention away from COVID-19 prevention and control measures, additionally exacerbating the pandemic situation [34]. Consequently, there was a remarkable increase in COVID-19-related fatalities [33]. The healthcare system was forced to prioritize the management of war-related injuries, placing additional strain on already limited resources and capacity for COVID-19 diagnosis and treatment [34]. 

No comprehensive assessments were conducted to explore the disruption of PHC essential services in Armenia. Understanding the extent to which those services suffered due to COVID-19 allows for better evidence-based decision-making and preparedness during future emergencies and pandemics. Moreover, understanding the reasons, most impacted areas, and nuanced nature of underutilization of those services from various perspectives (such as providers of PHC essential services and regular visitors to healthcare facilities), is critical for developing recommendations and targeted interventions. Thus, this study aimed to explore the impact of the COVID-19 pandemic on the utilization and delivery of PHC essential services in Armenia.

## Methods

### Study design

We applied a conventional qualitative study design using semi-structured in-depth interviews (IDIs) to explore the experience of PHC essential services provision and utilization during the COVID-19 pandemic from various stakeholders’ perspectives. The qualitative design gave an opportunity to thoroughly investigate the underlying dimensions of the main issues and obtain rich data about the participants’ experiences regarding the continuity of essential healthcare services.

### Study settings, population and sampling

The study participants were recruited throughout Armenia including Yerevan (the capital city) and the regions (marzes). Through purposeful sampling technique, the research team targeted three groups of participants: (1) physicians providing PHC essential services such as endocrinologists, gynecologists/obstetricians, cardiologists, and pediatricians, (2) beneficiaries of PHC essential services such as patients with chronic heart disease, diabetes, hypertension, chronic obstructive pulmonary disease, chronic orthopedic condition, and mothers of children receiving immunizations, and (3) health policymakers. We employed a purposive sampling approach and recruited the PHC providers through the administration of their corresponding PHC facilities. The remaining participants were contacted directly through the social and professional network of the research team.

### Study instruments

The research team developed three semi-structured interview guides for this study to deeply explore participant perspectives with specific considerations for each group of participants (Supplementary Material 1). The development of questions was guided by the WHO interim guidance “COVID-19: Operational guidance for maintaining essential health services during an outbreak” [35] and “Continuity of essential health services: facility assessment tool.” [36] Both tools were developed in 2020 in response to the COVID-19 pandemic, and aimed to assist health systems in identifying challenges to maintaining the continuity of essential health services. The items from these tools were adapted and transformed into open-ended questions.

The interview guide was initially developed in English, translated into Armenian and pre-tested. Based on the experience of the initial IDI, the interview guide was revised, with improvements to the flow and formulation of the open-ended questions. A separate form was developed to record the participants’ demographic information (age, gender, region etc.) and to take field notes.

### Data collection

We conducted data collection from summer to fall 2021. To build rapport between the interviewers and participants, and to ensure credible responses, the physicians were interviewed by a pediatrician and public health specialist, interviews with PHC beneficiaries were conducted by a social worker and a public health specialist. The format of the IDIs were online and in person, according to the participants’ preferences. All of the online calls were video assisted to foster rapport building. Data collection was stopped after reaching data saturation.

Overall, 17 IDIs were conducted: nine IDIs among physicians providing essential health services; six IDIs among regular visitors of PHC facilities; and two IDIs with policymakers. The mean duration of the IDIs was 42 min. The PHC physicians included two endocrinologists, two gynecologists/obstetricians, one cardiologist, and one pediatrician. The PHC regular visitors interviewed in this study were on average 58 years old, ranging from 25 to 75 years. Most of the PHC regular visitors were women (five out of six) and from Yerevan (five out of six).

### Data management and analysis

The IDIs were audio-recorded with consent from each participant. In cases where the participants refused to be recorded, interviewers took detailed written notes. All of the IDIs were transcribed verbatim in Armenian (the native language). Iterative thematic analysis was performed, coding the textual data and categorizing based on inductively emerging categories. Two research team members (one of them was the interviewer of the corresponding IDI) performed the coding of the transcripts. Weekly team meetings were arranged to discuss and agree on the coding and the identified patterns.

The Institutional Review Board #1 of the American University of Armenia approved the study protocol (#AUA-2021-009).

## Results

As guided by the emerged data, we grouped the study findings into five main categories: mobilization and organization of PHC services during COVID-19; PHC visits during COVID-19; worsening of chronic conditions due to the decline in PHC visits; problems with routine childhood vaccinations; and patient-provider communication challenges.

### Mobilization and organization of PHC services during COVID-19

The interviewed physicians had varying opinions regarding the impact of COVID-19 on the PHC system in Armenia. A few physicians highlighted some positive effects, stating that PHC became more organized and mobilized its resources better.

It [COVID-19] made healthcare in Armenia become more organized, responsive and mobilized. Physician IDI-4, Endocrinologist.

According to gynecologists, all services provided to pregnant women at PHC facilities continued with one modification, the scheduling of appointments. They explained that during the waves of the pandemic, planned and scheduled visits came to replace what had existed up until then - unplanned, on-demand visits, which occurred at the “patient’s convenience.”

Now a pregnant woman does not come whenever she wants. I examine the pregnant woman, the next visit is scheduled according to her gestational age, she comes on that [scheduled] day. Physician IDI-1, Gynecologist.

Essential pediatric services in the PHC facilities were also modified. Most PHC facility administrations limited pediatric visits and screenings to the most essential ones, such as screenings under the age of one year.

We reduced the number of screenings to a minimum, so that there were no “healthy” visits and the flow of healthy kids to the facility was minimized and healthy children did not meet each other [in the PHC facility]… Physician IDI-3, Pediatrician.

The frequency of the pediatric home visits was also adjusted in response to COVID-19: before the pandemic, pediatric home visits were performed on an as needed basis even for mild to moderate symptoms, while during the pandemic waves those were performed only if absolutely necessary based on medical indications.

Home visits were performed only if absolutely necessary, based on the medical indication… meaning [home visits] were not performed for just a sneeze, or for a case where [a child had] a temperature of 37^°^C. Physician IDI-3, Pediatrician.

One pediatrician explained that the type of patients, in terms of severity and nature of complaints, did not change significantly. However, the number of pediatric visits decreased due to a slight decrease in other infectious diseases among children, explained by widely practiced social distancing, hand hygiene and masks.

…the visits by type of chief complaints stayed the same, meaning they [who had complaints] were coming as usual, just the number of those having complaints decreased because they [children] didn’t socialize, they were mostly isolated at home and didn’t get sick… Physician IDI-3, Pediatrician.

These findings were also triangulated with mothers who were interviewed. They specified that COVID-19 served as a restraining factor for visits to PHC facilities. Additionally, PHC facilities introduced some restrictions on the number of accompanying adults during the visit.

I’m an anxious mom, I can take my child to a doctor even for the smallest thing, but in this COVID situation, it [COVID-19] was restraining me… Only my child and I were allowed [to enter the PHC facility]. Despite the challenge of being alone with a child in the polyclinic nobody else was allowed [to accompany us]. Visitor IDI-3, Mother.

### PHC visits during COVID-19

Most of the interviewed participants, including policy makers and physicians reported a decrease in PHC visits.

The statistics show that we clearly already have an attendance problem. Policymaker IDI-1.

The total number of patients decreased significantly during that wave. Sporadic patients came in case of extreme need, let’s say, when referrals were needed for hospitalization. Physician IDI-5, Cardiologist.

According to some physicians, the decrease in PHC visits was in part due to fear of contracting COVID-19 due to lack of information, and misinformation in the public, resulting in panic. Some of the physicians tried to take control of the situation through proactive counseling and used leaflets produced by the Ministry of Health (MoH) for that purpose.

You try to calm them down, explain or at least guide them to read [materials] from the right sources so that there is no pointless panic, no fear…we also tried [to inform] in the form of booklets, for example, about breastfeeding recommendations during COVID-19 for pregnant women and mothers. Physician IDI-3, Pediatrician.

According to some patients, the need for health services was a higher priority than the risk of getting infected with COVID-19, however, the majority described avoiding visits to the PHC facilities.

Well, now during the COVID-19 period… I have to get medication. So if I don’t go, I won’t get medication. I must go, sign, get it. Visitor IDI-1.

This avoidance of PHC visits was mainly observed during the pandemic waves and was explained as a change in health-seeking behavior of patients driven by public fear of contracting COVID-19 and partly by quarantine restrictions. Furthermore, several patients explained that they avoided visits to the PHC facilities even when the visit was to pick up free medications, preferring to buy the medications from a pharmacy as a “less risky” alternative.

We haven’t visited the [PHC facility] since COVID started. We limited our in-person visits. We have a pharmacy nearby, more often he [the patient’s husband] bought the medicine himself, and we didn’t go to the [PHC facility] to get it for free. Visitor IDI-5.

### Worsening of chronic conditions due to the decline in PHC visits

The decline in PHC visits eventually led to negative consequences in terms of the health of chronic disease patients. According to the physicians interviewed, the provision of PHC services to patients with chronic conditions suffered significantly during the pandemic. They highlighted that COVID-19 related restrictions caused challenges in follow-up and monitoring of patients with chronic conditions.

Our service requires constant contact with the patient, which [constant contact] is a must, and it has been greatly impacted. Physician IDI-6, Endocrinologist.

All PHC physicians noted that the severity of chronic conditions changed, noting more decompensated or exacerbated cases of diabetes mellitus, chronic heart disease, hypertension, and gynecological issues. Physicians felt that these changes in severity were a result of irregular visits and inconsistent follow-up, as well as related to COVID-19 disease itself.

Our chronic patients started to visit much less frequently; they were afraid to come to a medical facility. It contributed to the deterioration of their health status… Physician IDI-5, Cardiologist.

Gynecologists emphasized that the decrease in routine gynecological care led to a decline in “healthy woman” (preventive) visits, which subsequently resulted in the deterioration of patients’ conditions and some cases reached a critical state. This decline in preventive care contributed to missed opportunities for early detection and prevention of diseases that could have been otherwise addressed.

Gynecological visits decreased very sharply. It was only in extreme cases, when it was already a severe and an unbearable condition, only then they [patients]did consult a doctor. Physician IDI-2, Gynecologist.

The PHC physicians specified that no COVID-19 specific changes were made to chronic patients’ management guidelines, and some physicians considered this to be a gap. While most of them found there was no need for any specific modifications, a few providers (a cardiologist and a gynecologist) felt that lack of knowledge and preparedness was a major challenge. For example, cardiologists pointed out insufficient preparedness and lack of confidence in the pharmacological management of cardiac patients with COVID-19 disease due to lack of familiarity with the safety profile and side effects of several medications that were used for COVID-19 treatment.

…there were some medicines, that we, the primary unit [PHC facility], were prescribing with a little hesitancy, there was a fear that the patient could have complications. You know, first of all, we were not very familiar with that drug, [we] didn’t have much information about it. Physician IDI-5, Cardiologist.

Similar thoughts were shared by the gynecologists, who felt that little was known about COVID-19 disease in pregnancy so far, and that COVID-19 being “unknown” and “unpredictable” made their work even more difficult.

Nowadays any pregnancy complication or bad outcome is assumed to be related to COVID…as we don’t know much, whatever happens, we might blame COVID… Physician IDI-1, Gynecologist.

PHC physicians providing care for chronic patients did not receive any specific training, or received late trainings, about COVID-19 considerations for their respective areas of specialty. The MOH guidelines were considered enough by the majority of interviewed physicians. A few of them didn’t attend any trainings at all.

No, I did not undergo any special training, I was not personally called to trainings. Perhaps it wasn’t necessary as a subspecialist, but I am, well, familiar with it [COVID-19 and specifics related to endocrinology]. Physician IDI-6, Endocrinologist.

Almost all physicians mentioned mass media (e.g. television, social media) as their main source of information to get updates on COVID-19. All of them highlighted the lack of targeted and timely trainings as a major challenge to providing quality services during the pandemic. Some of the participants attended webinars and trainings through their own initiative by using personal contacts.

### Postponed routine childhood vaccinations

According to one pediatrician, there was a decline in all vaccine administrations in the beginning of the pandemic due to fear of contracting the virus. Eventually, the majority of those who had refused to receive vaccinations did receive them, about 2–3 months late. Pediatricians felt that the true vaccine refusal rates due to vaccine hesitancy during the pandemic waves didn’t actually differ from the pre-pandemic period.

They are compliant moms, they’ve [their children] received [vaccines]until now, they just constantly postponed it because of fear, but those [who postponed] were a very small percentage. It [the percentage of those who postponed] was higher at the beginning, during the first 6–7 months of COVID; afterward, they continued [to get vaccinations] as usual. Physician IDI-3, Pediatrician.

These findings were triangulated between the interviewed physicians, mothers, and policymakers. One of the mothers mentioned that she followed the national immunization schedule exactly as it was written for her child and took the child to the necessary medical check-ups. Another mother mentioned delays in scheduled immunizations.

Well, basically the process of immunizations continued. Policymaker IDI-1.

It [COVID-19] was at its peak in May when my newborn turned 45 days old, we were very stressed and scared. I consulted [with the pediatrician] myself, and s/he said, well… since you don’t go out much, let’s delay [the vaccination] a bit. Visitor IDI-6, Mother.

### Patient-provider communications

Overall, the usage of digital technologies for ensuring continuous patient-provider communication did not go beyond phone calls. Even though the patients with chronic conditions decreased their PHC visits, some providers continued to monitor them via phone calls. The latter caused an additional burden for the physicians.

Via phone calls… It was very hard… we received so many phone calls during different hours of the day, we answered calls until 11–12 at night. Physician IDI-1, Cardiologist.

Telephone communication was especially useful and frequently used between pediatricians and mothers.

We would talk on the phone and decide whether a home visit is necessary or not. Physician IDI-3, Pediatrician.

In other [non-urgent] cases, [the pediatrician] told me over the phone what I should do and how. I would call the doctor [child’s pediatrician], if s/he felt it was serious s/he would say [to visit the physician], in other cases she told me what to do over the phone. Visitor IDI-3, Mother.

Other types of communication, such as Viber, WhatsApp, Zoom, etc., were used infrequently for patient-provider communications. Endocrinologists continued monitoring patients not only through phone calls, but also by speaking or meeting with the relatives of patients, who would visit instead of the patients in those cases where patients could or would not visit the facility themselves (due to fear, illness, or other unspecified reasons).

I didn’t use services like telemedicine, etc. [I followed up] only by calling patients: if I had a patient to whom I prescribed follow-up, I was calling myself once or several times a day… Physician IDI-6, Endocrinologist.

Though not widespread, in regional PHC facilities, Zoom application was useful for providing parenting and motherhood trainings to pregnant women.

For example, we used to have a Motherhood School, 2 days… we met on Saturdays, Sundays, or even on weekdays, we used to organize a meeting with pregnant women… Now with this pandemic we are doing it online, now we can’t actually assemble pregnant women for the school, but we do the training online via Zoom… Physician IDI-2, Gynecologist.

## Discussion

This study aimed to investigate and gain insights into the utilization, transformation, and maintenance of PHC essential services in Armenia during the COVID-19 pandemic. The study findings revealed that during the pandemic, most of the essential services provided in PHC facilities continued with decreased in-person visits; this decrease was related to modification of the provision of services, lockdown restrictions, and public fear of visiting healthcare facilities. During the pandemic waves, maternal and child health services changed mostly in terms of the scope and scheduling of visits. Specifically, planned visits for pregnant women with appointment times came to replace the previously existing unscheduled visits that occurred at the “patient’s convenience.” Moreover, providers noted that there were no concerns about health-seeking behaviors of pregnant women as they continued to utilize essential services and were constantly in touch with their physicians via telephone. A study conducted in the Netherlands showed similar changes in antenatal care services such as decreased in-person visits and increased use of digital technologies, particularly telephone counseling [37]. However, many studies conducted in different countries worldwide during the pandemic reported lack of access, delays, and disruptions in antenatal care resulting in increased maternal and neonatal mortality [38–41]. A systematic review and meta-analysis of 40 studies exploring the effects of the COVID-19 pandemic on maternal and child health services concluded that, globally, these services were significantly impacted with worsened maternal and child health outcomes, such as increased number of maternal deaths, stillbirth, complicated pregnancies, and maternal depression [20]. 

The study participants explained that pediatric services underwent some modifications and were kept to the minimum essential to avoid unnecessary in-person visits, however, no major disruptions occurred. Additionally, increased use of telephone communications (e.g. phone calls and texting) was noted. Similarly, a study conducted in Germany showed that the number of pediatric consultations, particularly scheduled developmental examinations at the PHC level, remained relatively stable during the early stages of the pandemic [42]. In contrast, several studies reported major disruptions in pediatric health services worldwide, resulting in poor health outcomes including malnutrition, increased hospitalizations, morbidity, and mortality [43, 44]. According to the pediatricians interviewed in this study, the number of pediatric visits also declined because of the natural secondary effect of the widely practiced infection prevention and control measures by the public (that in its turn decreased other infections among children, reducing the medical need for visiting PHC facilities). A study conducted in Italy showed that public health interventions and infection prevention and control measures implemented during the COVID-19 pandemic reduced other respiratory viruses and related diseases among children [45]. 

Studies reported a worldwide decline in all pediatric vaccine uptake during the COVID-19 pandemic, and the WHO and UNICEF (United Nations Children’s Fund) declared this as the “largest continued backslide in vaccinations in three decades.” [46] However, according to our study participants, the true vaccine refusal rates during the pandemic waves were not perceived different from the pre-pandemic period and routine childhood vaccinations continued with slight delays in their administration. This might be explained by the fact that the population of Armenia is mostly compliant with the vaccination recommendations and the coverage in Armenia has been above 90% for the last decade [47]. Delays in routine vaccinations among children were also observed in studies conducted in many different countries [48–51]. As in other middle- and high-income countries, the main reason behind such delays was fear of visiting PHC healthcare facilities triggered by patients’ perceived fear of contracting the virus while in the facility [48–51]. A study conducted in Japan revealed that delayed vaccinations among children were later caught-up [48]. Interventions should continuously focus on preventing vaccination delays and helping children catch up on vaccinations to prevent the spread of vaccine-preventable diseases in Armenia. For example, a study conducted in the United States showed that there was a lag in catching up with the delayed routine vaccinations that could result in an increased number of vaccine-preventable diseases, posing a serious public health concern [49]. 

An encouraging finding was that the supply of essential medications to patients with chronic conditions was successfully maintained with no disruptions, even during the lockdowns. This was managed not only by the government, but also by efforts of individual PHC facilities. As an example of the latter, in some cases, nurses delivered medications to patients with chronic conditions, together with other social support packages during the pandemic waves, when patients avoided visiting PHC facilities because of the fear of being infected. Such adaptation and transformation of care delivery systems have also been described in the literature [52–54]. However, given how overwhelmed government structures and healthcare systems become during pandemics, such adaptations might not always be feasible to organize.

Similar to previously reported studies, the utilization and provision of PHC essential services for patients with various chronic conditions suffered significantly [12, 17, 27]. The reported remarkable decrease in patient visits with chronic conditions for both routine and follow-up visits was perceived to be the result of negatively changed health-seeking behavior of patients driven by the fear of being infected with the SARS-COV2, and in some settings by the quarantine restrictions. These behavioral changes were also reported across other countries independent of the country income level and health system structure [54–56]. The study findings also showed that despite increased telephone communication between physicians and patients, irregular visits and lack of close follow-up had “expected” effects on worsening of chronic conditions. This particularly led to worsened conditions of patients with diabetes and chronic heart diseases. Studies conducted in many countries reported similar experiences [57]. Moreover, disruptions in PHC service utilization for this subset of the population have long-term consequences such as complications of unregulated chronic diseases and severe life-threatening conditions (e.g. diabetic foot, peripheral vascular disease, myocardial infarction, stroke, and others) [57]. These findings are of utmost importance, considering the already known risk of severe illness and excess death from COVID-19 in these high-risk populations [2, 3, 11, 16, 58]. 

One of the evidence-based solutions that could assist in maintaining proper services at PHC level is telemedicine. It is an effective measure for patient-provider continuous contact at the PHC level [57]. Studies show that it improved the quality of life and reduced morbidity and mortality among patients with chronic diseases [57]. In light of the COVID-19 pandemic, telemedicine was given a boost and was extensively used globally to reduce in-person visits and maintain essential health services without compromising quality [59]. Telemedicine was especially relevant and effective in the provision of sustainable services for patients in low-resource settings [60, 61]. In line with the international literature, this study also showed that phone calls were the main method of patient-provider communication during the pandemic at the PHC level [62, 63]. However, there is no specific data on the number of phone calls made or the proportion of in-person visits replaced by telephone calls. This missing information is important as it would help to determine the extent to which phone calls served as substitutes for missed in-person visits. The use of other telemedicine modalities (e.g. videoconferencing platforms) was limited. Studies showed that even though video communication was the preferred mode of patient-provider contact (e.g. when visual assessment is needed), it had some disadvantages in terms of accessibility and feasibility [64, 65]. Our study did not specifically address the ease of use or accessibility of video visits. Factors such as limited training, inadequate infrastructure, and patient unfamiliarity may contribute to the lack of use in video communication. Efforts should be made to address the barriers mentioned earlier and improve training, technology access, and patient education. This optimization of video visits in PHC settings is particularly important during crises like the COVID-19 pandemic. The level of utilization of telemedicine at the PHC level in Armenia needs to be further explored and explained. Studies must identify existing gaps, challenges, and opportunities for future implementation and standardization of telemedicine for ensuring the continuous provision of PHC essential services and avoiding exacerbated cases of chronic conditions. Moreover, countries with similar experiences should develop mechanisms to register, monitor and compensate healthcare providers for their consultations using digital technologies.

Our findings revealed that PHC providers experienced challenges related to their lack of preparedness on the management of chronic conditions during the pandemic. This was particularly evident when these conditions were accompanied by COVID-19 infection, especially during the first wave of the pandemic. Moreover, they had difficulties in counseling their patients, because the overall situation was driven by panic, and visits were frequently missed. These gaps could be resolved by targeted trainings, availability of up-to-date practice guidelines, and with more structured and strategic use of telemedicine.

### Strengths and limitations of the study

This study was a comprehensive assessment of PHC essential services during the pandemic using a conventional qualitative study design and semi-structured in-depth interviews. Trustworthiness of the study was fulfilled through widely accepted criteria such as credibility, dependability and confirmability [66]. We involved physicians and patients from both public and private facilities, ensuring data-source triangulation of the findings and, thus, credibility. We also conducted iterative thematic analysis, transcription and analysis in the original language, which ensured that interpretations and analysis of the findings were clearly derived from the data, without content changes due to translation. The coding process was done by two members of the research team and cross-validated with a third researcher. However, the study has several limitations. Due to time constraints and COVID-19 related restrictions, we were unable to conduct all interviews face-to-face, and were not able to engage more participants from rural settings, which might limit the generalizability of the findings. We did not include nurses of PHC sector in this study who could provide different perspectives. Additionally, although the bias of leading questions was minimized in this study by using open-ended and neutral questions, there is still some risk of social desirability bias on the part of PHC providers.

## Conclusion and recommendations

This study demonstrated that the COVID-19 pandemic has affected the delivery and utilization of essential healthcare services at PHC facilities, and informed health policy makers to make targeted interventions and strengthen PHC capacity to respond to future pandemics. Importantly, the study showed that the pandemic significantly impacted visitors of PHC facilities with chronic conditions who needed regular follow-up. This suggests that for maintaining continuous provision of PHC essential health services to vulnerable populations, better guidance and technical capacity is needed. A unified national-level action plan for PHC facilities is needed to respond to potential outbreaks and continue effective provision of essential services and health communication. PHC facilities must be equipped and providers trained on the use of various digital platforms. Efforts are needed to develop effective health and risk communication strategies and enhance the effective use of digital platforms to provide healthcare services and promote appropriate health-seeking behaviors among the public.

### Electronic supplementary material

Below is the link to the electronic supplementary material.

Supplementary Material 1

## Data Availability

The data used and/or analyzed during the current study are available from the corresponding author on reasonable request.

## Abbreviations

COVID-19: Coronavirus disease 2019
PHC: Primary healthcare
LMIC: Low- and middle-income country
WHO: World Health Organization
IDI: In-depth interview
UNICEF: United Nations Children’s Fund
SARS-COV2: Severe acute respiratory syndrome coronavirus 2
