# Supplementary material for: Impact of COVID-19 on essential healthcare services at the primary healthcare level in Armenia: a qualitative study
Source: BMC Prim Care. 2024 Apr 24;25:131. doi: 10.1186/s12875-024-02377-6 (PMC11044568; doi:10.1186/s12875-024-02377-6)
Supplement: Supplementary file 1 — Supplementary Material 1 [file 12875_2024_2377_MOESM1_ESM.docx]

**SUPPLEMENTARY MATERIAL**

**INTERVIEW GUIDES**

**IDI guide for PHC facility regular visitors**

**Let us talk about the PHC service utilization and your experience during COVID-19.**

1. Could you please tell us about your experience of using PHC services during Covid-19 period? If you visited the polyclinic, what was the reason for the visit? PROBES: *What PHC services did you use?*
2. Did you notice any differences in PHC service delivery/organization before and after COVID-19 period? How would you describe those differences?
3. How COVID-19 situation afffected the frequency of your visits to PHC facilities to receive health services? PROBES: *How the frequency of your visits to PHC facility was increased or decreased and what are the reasons? Please describe what services did you start using more or less as compared to before COVID-19 times? Have you ever hesitated utilizing health services due to COVID-19, please explain why?*
4. How did you obtain essential health services from PHC? PROBES: *Please describe what remote services/digital solutions (calls, SMS, video-calls) or other means/strategies you were offered during COVID-19 pandemic to limit facility based encounters?*
5. Please describe any disruption of the services provided by your PHC facility during COVID-19? **PROBES:** *Have you been denied for any services due to COVID-19 situation? Were there postponed/suspended services/ missed appointments due to COVID-19? How the suspended services or missed visits/appointments were addressed?*
6. Please describe how did you receive the necessary medications and supplies required for your chronic disease management from your PHC facility (if applicable). Please compare your experience before and during COVID-19 pandemic.
7. How satisfied were you with the polyclinic services before and during COVID-19 situation? What would you improve?
8. Please describe what infection prevention and control (IPC) measures have you used when visiting your PHC facility during COVID-19 pandemic? What IPC measures were taken by the PHC facility during your visit?
9. Please tell us how you got informed about IPC measures? PROBE: *What was the role of your doctor or the PHC facility in informing/educating you about IPC*. *Other than being informed from your doctor/polyclinic, what other sources of IPC information did you encounter?*
10. Overall, how satisfied were you with the IPC measures taken in the polyclinic or instructed to you by your doctor? What would you have added to the instructions about IPC given to you by your doctor?
11. How would you describe the support received from your doctor or PHC facility in order to continue using necessary health services during the outbreak such as the services you usually utilize.
12. How have you been informed about the changes in service delivery during COVID-19 pandemic at your PHC facility?
13. Please tell us about your overall impression of your experience with health service delivery at your polyclinic during COVID-19 pandemic. What were your main concerns and obstacles? What would you recommend to improve in the polyclinic to better meet your needs during pandemic?

**IDI guide for cardiologists, endocrinologists and OB/GYN**

**Let us talk about the service provision and your experience during COVID-19.**

1. How was health services provision affected during COVID-19 (*PROBES:* ***cardiologist:*** *Diagnosis and treatment of chronic cardiovascular disease;* ***endocrinologist:*** *Diabetes screening, diagnosis and treatment)?*
   1. ***OB/GYN:*** *Cancer screening, diagnosis and treatment)*?

How sexual and reproductive health services provision was affected during COVID-19 at PHC level? *PROBES: Family planning and contraception; Antenatal care; Postnatal care; Immunization services; Prevention, diagnosis and treatment of sexually transmitted infections; Intimate partner and sexual violence –prevention and response.*

1. Were the patient volume and patient type changed during COVID-19?
2. How would you describe/explain the likely reasons for increased or decreased outpatient attendance for certain services?
3. Has there been any disruption of the services provided by the facility during COVID-19?
4. Were there postponed/suspended services/ missed appointments in your practice due to COVID-19? How the suspended services are anticipated to restore based on the changing needs of public health? How were the missed visits/appointments addressed? Are there any plans to catch up (*PROBES:* ***cardiologist:*** patients with chronic non-communicable diseases/CVD patients; ***endocrinologist:*** patients with chronic non-communicable diseases/diabetes; ***OB/GYN:*** for pregnant women)?
5. How was your work affected by the COVID-19? What were the main reasons for your absence (if any) during COVID-19 period?

**Now let us talk about the strategies to limit facility-based encounters.**

1. What strategies were used to limit the number of facility-based encounters?
2. How available technologies were used to facilitate the shift of clinical encounters to digital platforms and support self-care interventions wherever appropriate?
3. What digital health tools have been implemented to maintain and strengthen the delivery of essential health services and communicate to the public about how to access these services? How were these platforms/tools chosen? *PROBES: telemedicine, sms-messaging to ensure medication adherence, to deliver self-care interventions etc.*
4. How the chronic disease management strategies were redesigned around limited or adapted provider encounters and increased self-management, while ensuring access to necessary medications and supplies?
5. What kind of trainings (if any) did you receive on the use of digital technologies? Are there any guidelines available to you?

**Now let us talk about your received training and IPC measures.**

1. How were you trained or supported related to COVID-19? What kind of training or support have you received? *PROBES: Training on infection prevention and control (IPC); Training on proper use of personal protective equipment (PPE); Mental health and psychosocial support; Supportive supervision for IPC; Supportive supervision on proper use of PPE; Training on provision of remote health care; Training on digital tools.*
2. Does the facility have IPC guidelines for COVID-19? How were you trained regarding IPC?
3. How were the adequate IPC supplies ensured in the PHC facility to guarantee the safe delivery of services? *PROBES: Gown, protective; Gloves, examination; Goggles, protective; Face shield; Respirator masks (N95 or FFP2); Mask, medical/surgical. How and when were you provided with adequate PPE supplies and appropriate IPC to protect yourself as a health care provider?*

**Now let us talk about public communication approaches.**

1. What communication approaches were used to build public confidence and encourage continued utilization of essential services during the outbreak such as your services?
2. How was the information disseminated to guide safe care-seeking behavior among your patients? How was the public prepared for changes in service delivery at your PHC facility?

**IDI guide for policymakers**

1. Please describe any changes that your PHC facility faced during COVID-19 pandemic?
   - Was the capacity reallocated from routine comprehensive services towards essential services?
   - Were the facilities been closed temporarily because of a COVID-19? Were there changes in working hours?
2. How would you describe the defined list of essential health services for your PHC facility before the COVID-19 pandemic?
   - How was this list of services changed or modified during COVID-19? PROBES: Reduced the scope of specific services; Reduced the volume of specific services; Suspended the provision of specific services; Redirected patients to alternative health care facilities, etc.
   - How the outreach health services were affected due to COVID-19 (e.g., home visits)?
   - How were the missed visits/appointments addressed? Are there any plans to catch up (e.g., for pregnant women, Children for routine immunization; Patients with chronic non-communicable diseases?
3. How was the workforce planning conducted? How the mapping of the need for healthcare workers is being conducted? Are there any plans for different COVID-19 transmission scenarios?
4. What were the main reasons for health workforce absence during COVID-19 period? Which healthcare providers are mostly affected (e.g., gender differences)?
5. How the facility received additional funding to ensure the maintenance of essential health services during the pandemic? What is the source of the additional funding?
6. Were there any adjustments or changes in salaries of all personnel, including staff and contractors? Were there changes in payment schedule (e.g., any delays)? Have any personnel worked overtime? Were they compensated for that?

*Now let us talk about the communication strategies for supporting the appropriate use of essential services.*

1. What communication approaches were used to build public confidence and encourage continued utilization of essential services during the outbreak? PROBES: such as primary care clinics, pharmacies, community health workers and leaders, and peer networks, etc.
2. How was the information disseminated to guide safe care-seeking behavior? How was the public prepared for changes in service delivery platforms (including outreach activities in their communities)?

*Let us talk about the IPC program in your facility.*

1. How is IPC coordinated in your facility?
   Probes: Is there a person responsible for IPC in your facility? (PROBES: What are the set of responsibilities of that person?)
2. What is the source of the budget for the IPC measures? Does it provide enough finances to cover the expenses, if no, what are additional sources? What are the measures taken for efficient spending?
3. Are there guidelines, SOPs that your facility follows? If yes, what kind of guidelines do you follow (national, facility level)? If there are no guidelines, how do you manage IPC at your facility?
4. What can you tell us about IPC Standard Operating Procedures (SOP) that you and your facilities’ personnel follow?

*I would like to discuss some questions about implemented environmental infrastructure measures to assure the IPC at the PHC level.*

1. What are the implemented environmental infrastructure measures to assure the IPC at PHC level (Water supply, sanitation, hand hygiene, waste management, ventilation, IPC supplies and equipment, disinfection of waiting/screening/ triage areas, PPE, decontamination and sterilization of supplies)?

*Now let us talk about the IPC supplies meeting the facility demand.*

1. How does the facility ensure that the IPC supplies’ demand is properly met?
2. Who is responsible for managing the IPC supply chain for necessary IPC supplies? What is included in his/her responsibilities? How is the IPC supply chain managed at your facility, if there is no responsible person for the job?
3. How would you describe the availability of vital IPC supplies necessary for suspected and/or confirmed COVID-19 patients (probe: How does your facility address supply shortages and monitoring consumption of IPC supply? Please describe the process)?

*Now, let us talk about the educational component of IPC.*

1. What would you tell us about COVID-19 IPC training and education of HC personnel as well as patients and visitors?
   What topics those trainings cover? How do you think the HC personnel is satisfied with these trainings? What would you improve?
2. In what manner is your facility involved in the awareness raising of patients about COVID-19 prevention and control? How do you ensure the IPC training and/or the education of the patients and visitors of your facility?
3. What are the measures taken for ensuring patient and health worker safety? (Probe: How do you assure the safety of patients in waiting areas? What measures are taken for assuring mask wearing and proper hand hygiene of the patients in the waiting room?
4. What challenges do you face in this process? What would you recommend to improve?

*Now let’s talk about the IPC assurance monitoring.*

1. How does the government monitor the assurance of structural and process indicators of IPC in your facility?
2. How do you monitor the proper assurance of IPC guidelines and SOPs within the facility? What IPC indicators do you monitor? How?
   (probe: compliance with hand hygiene, appropriate use of PPE, health worker infections, other)
3. What are the challenges in this process? What would you improve?
